# Atypical Presentation of Postoperative Guillain-Barré Syndrome in a Pediatric Patient: A Case Report and Review of the Literature

**DOI:** 10.7759/cureus.85672

**Published:** 2025-06-09

**Authors:** Chaymae Yechouti, Olfa Asbik, Ayyad Ghannam, Nadia Elmahi, Imane Kamaoui, Maria Rkain

**Affiliations:** 1 Pediatric Department, University Hospital Center of Mohammed VI, Faculty of Medicine and Pharmacy, Mohammed Premier University, Oujda, MAR; 2 Radiology Department, University Hospital Center of Mohammed VI, Faculty of Medicine and Pharmacy, Mohammed Premier University, Oujda, MAR

**Keywords:** electroneuromyography, guillain-barré syndrome, mri, neurological complication, post-surgery

## Abstract

Guillain-Barré syndrome (GBS) is an acute inflammatory polyneuropathy and a leading cause of acute flaccid paralysis in children. While commonly triggered by infections, GBS may rarely occur following surgical procedures. The pathogenesis of post-surgical GBS remains unclear, with immune-mediated mechanisms and perioperative factors being implicated.

We report the case of a 9-year and 10-month-old girl who developed GBS one week after surgical correction of a duodenal diaphragm. She presented with rapidly progressive tetraparesis, dysphagia, and respiratory distress in an afebrile context. Neurological examination revealed flaccid tetraparesis, abolished deep tendon reflexes, and no sensory or sphincter involvement. Electroneuromyography confirmed severe sensorimotor axonal polyneuropathy. Laboratory workup and imaging were unremarkable. The patient was treated with intravenous immunoglobulin and supportive therapy, resulting in favorable neurological recovery over time, with a stable condition at a three-year follow-up.

Postoperative GBS is a rare but serious complication, often underrecognized, especially in pediatric patients. This case underscores the importance of considering GBS in the differential diagnosis of acute neurological deficits following surgery. Prompt diagnosis and treatment are essential to improve outcomes.

Clinicians should maintain a high index of suspicion for postoperative GBS in the setting of acute neuromuscular weakness after surgery, as early immunotherapy can lead to favorable recovery.

## Introduction

Guillain-Barré syndrome (GBS) is an acute inflammatory polyneuropathy characterized by rapidly progressing, typically symmetric muscle weakness and absent reflexes in a previously healthy child [1]. GBS is the leading cause of acute flaccid paralysis in children. Its variable incidence across populations may be due to differences in genetic susceptibility or exposure to triggering pathogens [2]. This case report describes a pediatric patient who developed features of GBS seven days after undergoing surgery. The diagnosis was made based on the presentation of tetraparesis.

The precise pathophysiology of GBS following surgical procedures remains unclear. The most widely accepted etiological hypotheses involve immune-mediated mechanisms and perioperative infections. Given the rarity and diagnostic challenges of post-surgical GBS in children, further exploration of this association is important to strengthen the clinical relevance of this report.

## Case presentation

We report the case of a 9-year and 10-month-old girl with no significant past medical history and normal psychomotor and growth development. One month prior to her current admission, she presented with abdominal distension and was evaluated in the pediatric emergency department. A diagnosis of duodenal diaphragm was made, and she underwent surgical correction.

However, within one week postoperatively, her condition deteriorated, beginning with diffuse myalgia in both thighs, which rapidly progressed to complete functional impairment of the lower limbs. She subsequently developed upper limb weakness and myalgia, dysphagia, and tachypnea, all occurring in an afebrile context.

On clinical examination, she was conscious, with a Glasgow Coma Scale score of 15/15, asthenic, tachycardic at 128 beats per minute, and tachypneic at 35 breaths per minute. A neurological assessment revealed a peripheral neurogenic syndrome with truncal involvement, characterized by an inability to walk, flaccid hypotonia, muscle strength graded 0/5 in the lower limbs and reduced strength in the upper limbs, absent deep tendon reflexes, and indifferent plantar responses. There were no sensory or sphincter disturbances. The remainder of the physical examination was unremarkable, apart from a transverse laparotomy scar.

Laboratory investigations revealed a normal complete blood count, except for thrombocytosis at 717,000/μL. Electrolytes, inflammatory markers, vitamin levels (B1, B6, B9, B12), and creatine phosphokinase were within normal limits. Cerebrospinal fluid analysis showed no albuminocytological dissociation, as the lumbar puncture was performed on the third day after symptom onset - consistent with an early or atypical form of the disease.

**Figure 1:**
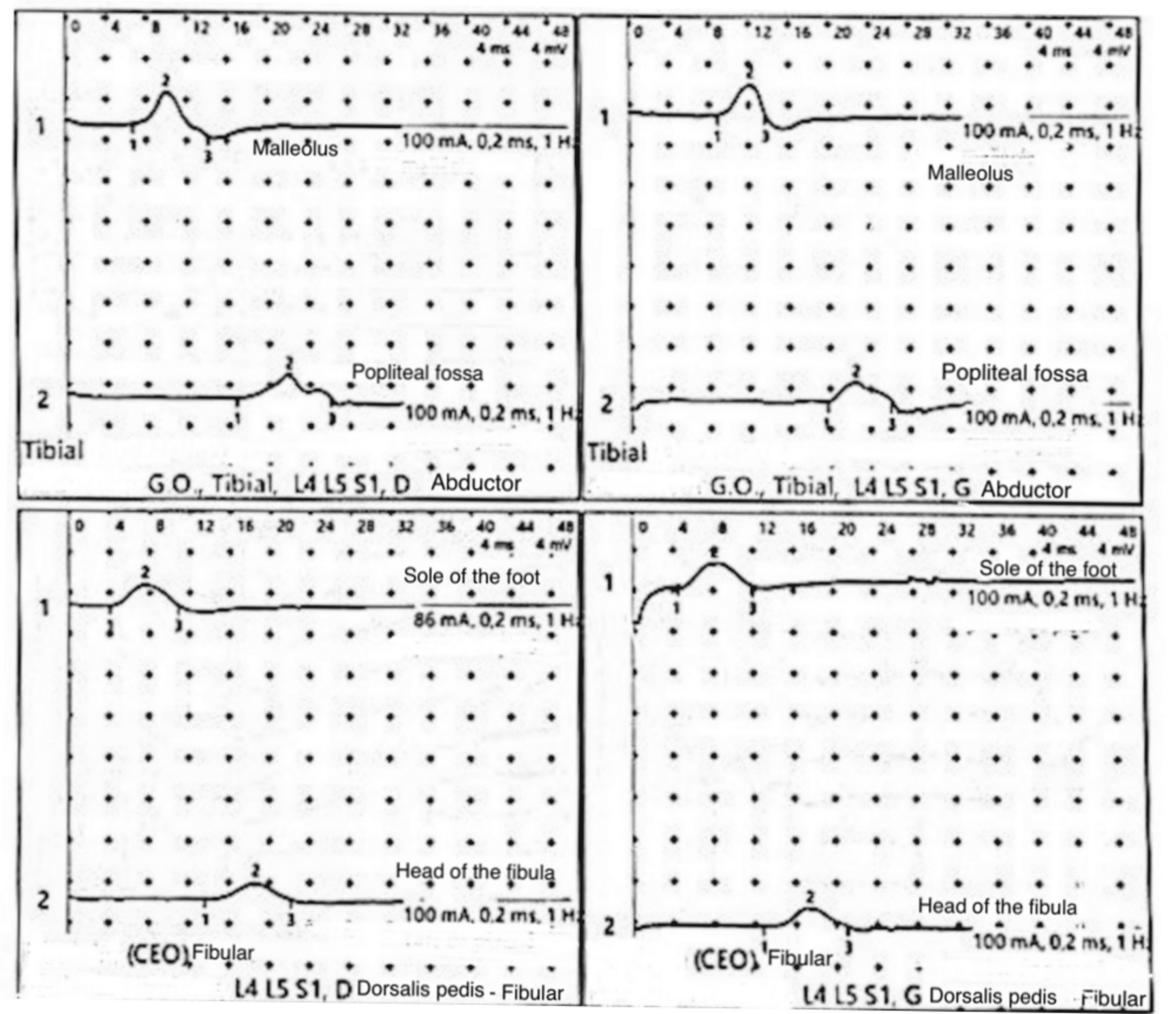
Nerve conduction study findings Motor nerve conduction studies of the tibial and peroneal nerves demonstrate bilaterally reduced compound muscle action potentials, prolonged distal motor latencies, and decreased conduction velocities. These findings are consistent with a symmetrical motor axonal neuropathy affecting the lower limbs. G.O.: gluteus origin

**Figure 2:**
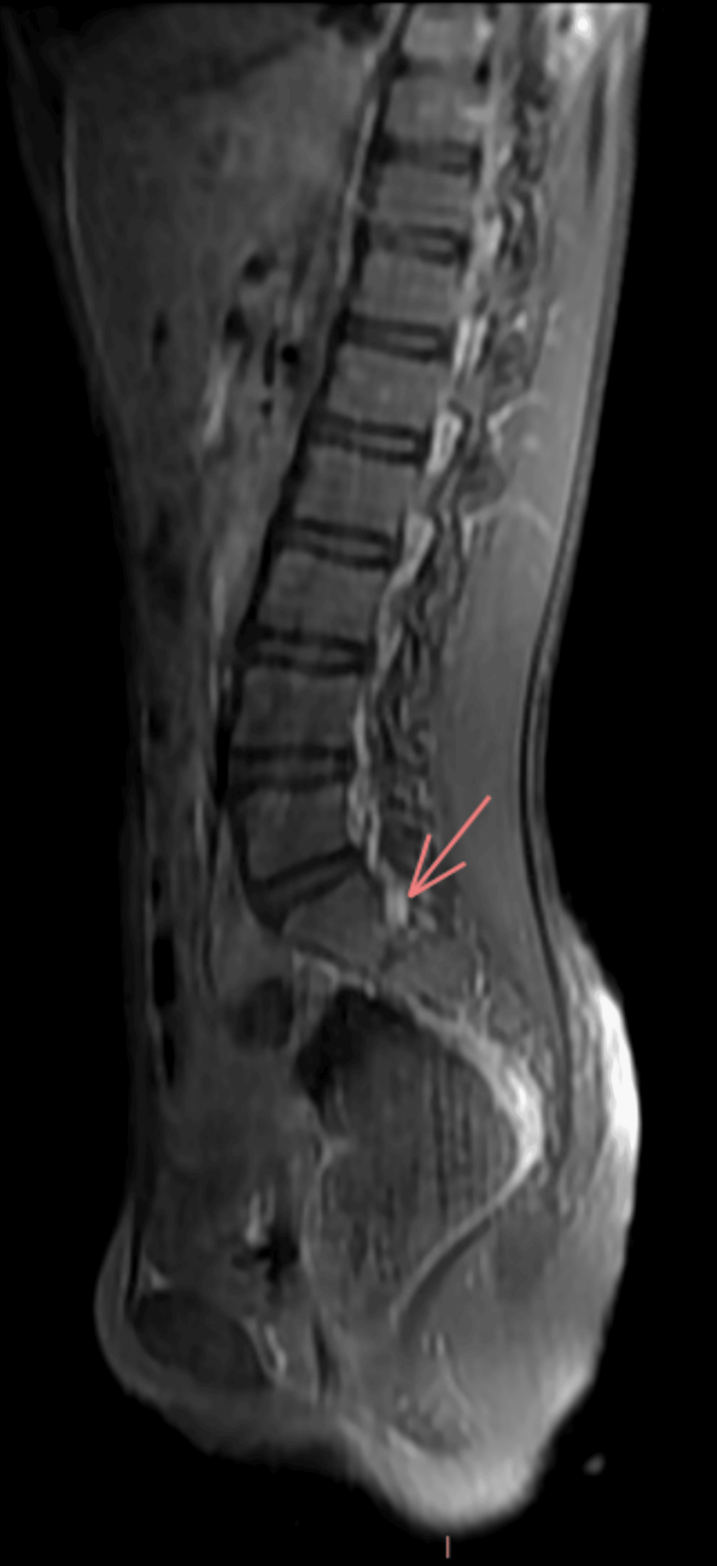
MRI with sagittal section in T1-weighted images with fat suppression and gadolinium contrast Brain and spinal MRI of our patient demonstrates homogeneous and diffuse thickening of the cauda equina, including the lumbar and sacral nerve roots, which appear swollen and show intense enhancement after gadolinium injection. The overall appearance is compatible with Guillain-Barré syndrome.

**Figure 3:**
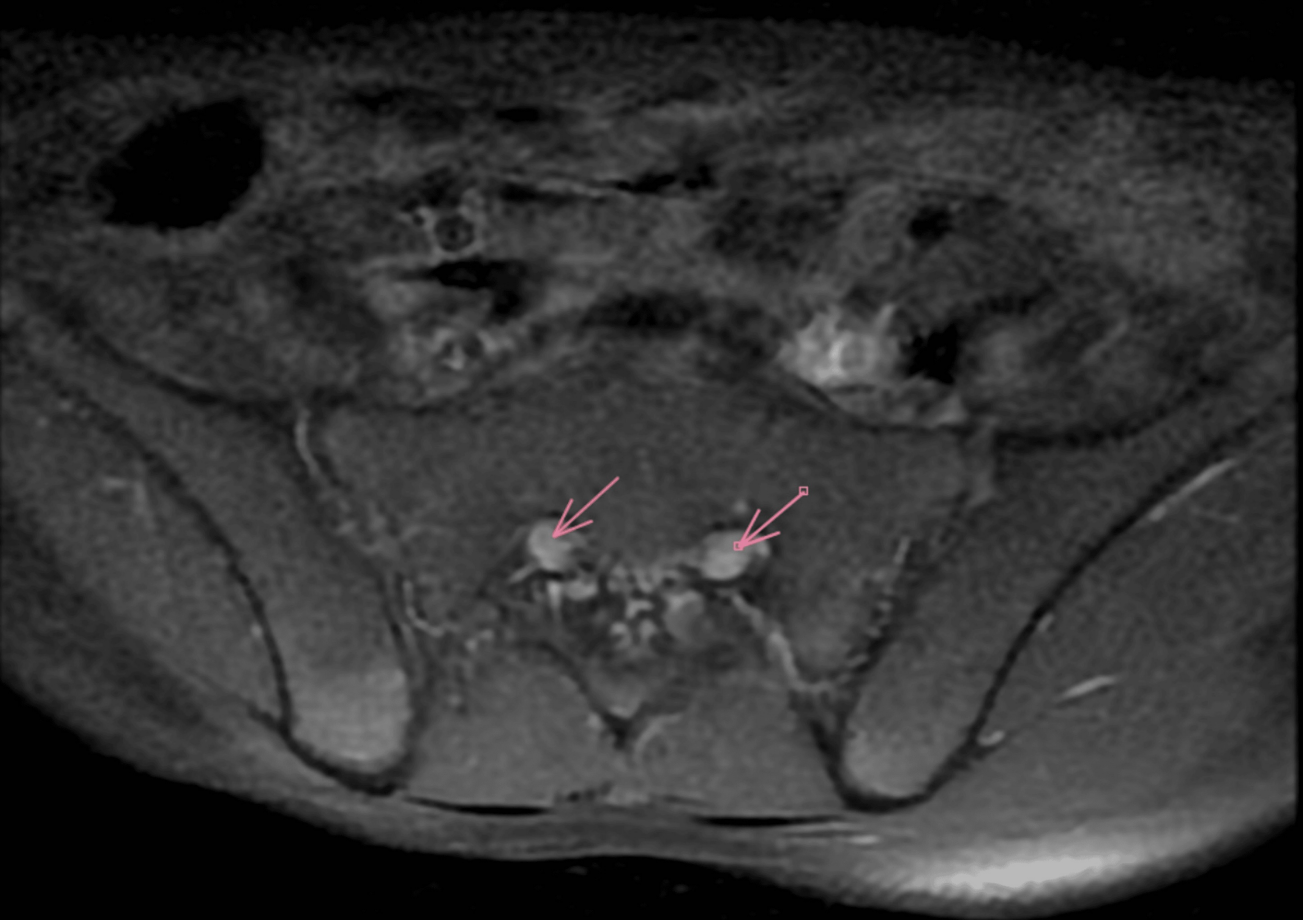
MRI with axial section in T1-weighted images with fat suppression and gadolinium contrast Brain and spinal MRI of our patient demonstrates symmetrical thickening of the lumbar nerve roots with marked enhancement following gadolinium administration. These findings are consistent with Guillain-Barré syndrome.

Electroneuromyography revealed a severe axonal sensorimotor polyneuropathy (Table 1, Figure 1). Brain and spinal MRI showed homogeneous and diffuse thickening of the cauda equina, including the lumbar and sacral nerve roots, which appeared swollen and demonstrated intense enhancement after gadolinium injection (Figures 2, 3). Based on the clinical and neurophysiological findings, a diagnosis of postoperative Guillain-Barré syndrome was strongly suspected.

**Table 1:** Results of motor conduction of the tibial and fibular nerves Electroneuromyography results demonstrate a symmetric motor and sensory axonal neuropathy, predominantly affecting the lower limbs. Nerve conduction velocities are reduced, with decreased amplitudes and prolonged latencies, compatible with axonal polyneuropathy. G.O.: gluteus origin

Nerve	Side	Target muscle	Stimulation point	Latency (ms)	Amplitude (mV)	Duration(ms)	Surface (mV·ms)	Stim (mA)	Dist (mm)	Speed (m/s)
Tibial	D	Abductor G.O.	Malleolus	6.7	3.2	7.49	8.6	100	320	35.6
Tibial	D	Abductor G.O.	Popliteal fossa	16.8	2.3	9.28	10.2	100	360	11.1
Tibial	G	Abductor G.O.	Malleolus	8.8	3.0	4.92	7.5	100	320	10.0
Tibial	G	Abductor G.O.	Popliteal fossa	19.7	2.0	6.48	6.8	100	390	10.4
Fibular	D	Dorsalis pedis	Sole of the foot	4.2	2.0	6.8	7.6	86	270	38.9
Fibular	D	Dorsalis pedis	Head of the fibula	13.7	1.6	8.56	10.1	100	270	2.84
Fibular	G	Dorsalis pedis	Sole of the foot	4.4	2.3	7.6	9.0	100	270	38.4
Fibular	G	Dorsalis pedis	Head of the fibula	12.9	1.7	8.24	6.2	100	370	8.4

The patient was treated with intravenous immunoglobulin at a dose of 1 g/kg/day for two consecutive days, in combination with supportive care, including analgesics, vitamin supplementation, and early physical rehabilitation. The clinical course showed moderate improvement by day four following IVIG administration, as evidenced by the patient's ability to stand and take a few assisted steps. By day 10, she regained autonomous ambulation, followed by progressive neurological recovery. At the three-year follow-up, the patient remained clinically stable, with no recurrence of symptoms.

## Discussion

Post-surgical GBS is a rare neurological complication. Its exact incidence remains uncertain due to variability in study cohorts and reporting methods. For example, one study reported 63 cases of post-operative GBS among 665 patients, yielding an incidence of 9.5% within six weeks following surgery - a relative risk 13.1 times higher than in the general population [1]. Although incidence rates vary between studies, these data collectively suggest an increased risk of GBS following surgical procedures.

GBS is typically associated with a preceding viral or bacterial infection. However, several documented cases have shown that some patients develop the syndrome after surgery without any identifiable recent infectious history. This suggests that surgery itself may act as a triggering factor, although current evidence remains limited [3-5].

The pathophysiological mechanisms underlying the onset of GBS after surgery are not yet fully understood. The most widely accepted hypothesis proposes that transient immunological stress induced by surgery may promote an autoimmune response, possibly via subclinical infection or molecular mimicry involving myelin proteins [6]. Certain surgical procedures may also expose peripheral nerve antigens, leading to an aberrant immune response.

The global incidence of GBS ranges from 0.3 to 6.6 cases per 100,000 persons per year, according to international cohort studies [1]. Post-surgical GBS is often under-recognized by clinicians. Early clinical signs, such as muscle weakness or numbness, may be misinterpreted as other postoperative complications, contributing to diagnostic delays. In our case, the diagnosis was made following the development of tetraparesis and absent deep tendon reflexes. Electroneuromyography confirmed a severe axonal sensorimotor polyneuropathy.

Recent evidence indicates that GBS has been observed following a variety of surgical procedures, including cardiac, neurosurgical, and spinal surgeries, as well as, less frequently, after regional anesthesia [7-9]. Reports of GBS following digestive or orthopedic surgeries are relatively rare. In our case, the patient developed GBS after diaphragmatic surgery, a scarcely reported occurrence, underscoring the uniqueness of this presentation.

Anesthetic agents, particularly those used during epidural anesthesia, may alter the local structure of myelin or compromise nerve integrity, thereby exposing myelin antigens to the host immune system [9]. This process is believed to stem from disruption of the blood-nerve barrier or from the direct neurotoxic effects of local anesthetics such as bupivacaine or lidocaine. When administered at high concentrations or over extended periods, these agents can lead to demyelination or axonal damage, resulting in the release of previously sequestered neural antigens. Furthermore, mechanical trauma from epidural needle insertion may provoke localized inflammation and immune activation, potentially triggering an autoimmune response in genetically or immunologically predisposed individuals.

Clinically, postoperative GBS typically presents with progressive, symmetrical muscle weakness in the limbs, accompanied by areflexia or hyporeflexia. Some patients may also experience sensory disturbances, including paresthesia, pain, or numbness [10-11].

Diagnosis relies on a comprehensive evaluation of the clinical context, surgical history, and electrophysiological studies. Differentiating GBS from other postoperative neuropathies can be challenging without the application of standardized diagnostic criteria [10].

Treatment is primarily based on immunotherapy, particularly the use of intravenous immunoglobulin (IVIG). IVIG exerts its therapeutic effects through multiple mechanisms, including modulation of the complement system, neutralization of pathogenic antibodies, inhibition of pro-inflammatory cytokines, and blockade of macrophage Fc receptors [12]. In our case, the clinical course showed moderate improvement by day four following IVIG administration, marked by the patient’s ability to stand and take a few supported steps. By day ten, autonomous ambulation was achieved, followed by gradual and sustained neurological recovery.

The overall prognosis is generally favorable, with neurological improvement typically occurring over a period of several weeks to a few months.

## Conclusions

Postoperative GBS is a rare yet serious neurological complication. It has been reported following various types of surgical procedures, including lung surgery in this instance. Physicians encountering unexpected neurological issues post-surgery should consider this diagnosis, which can be confirmed through NCS. Accurate diagnosis enables the initiation of proper treatment and monitoring, leading to recovery in the majority of cases.
